# Effects of Different Application Times of Silver Diamine Fluoride on Mineral Precipitation in Demineralized Dentin

**DOI:** 10.3390/dj9060070

**Published:** 2021-06-14

**Authors:** Surapong Srisomboon, Matana Kettratad, Phakkhananan Pakawanit, Catleya Rojviriya, Prathip Phantumvanit, Piyaphong Panpisut

**Affiliations:** 1 Unit of Gerodontology, Faculty of Dentistry, Thammasat University, T. Klong 1, A. Klongluang, Pathum Thani 12120, Thailand; surapong.sri@dome.tu.ac.th (S.S.); pmatana@staff.tu.ac.th (M.K.); 2 Synchrotron Light Research Institute (Public Organization), 111 University Avenue, Muang District, Nakhon Ratchasima 30000, Thailand; phakkhananan@slri.or.th (P.P.); catleya@slri.or.th (C.R.); 3 Faculty of Dentistry, Thammasat University, T. Klong 1, A. Klongluang, Pathum Thani 12120, Thailand; prathipphan@gmail.com; 4 Thammasat University Research Unit in Dental and Bone Substitute Biomaterials, Thammasat University, T. Klong 1, A. Klongluang, Pathum Thani 12120, Thailand

**Keywords:** silver diamine fluoride, dental caries, application time, demineralization, remineralization, mineral density

## Abstract

Silver diamine fluoride (SDF) is a cost-effective method for arresting active dental caries. However, the limited cooperation of patients may lead to an SDF application time that is shorter than the recommended 1–3 min for carious lesions. Therefore, the aim of this study was to assess the effect of different application times of SDF on the degree of mineral precipitation in demineralized dentin. Demineralized dentin specimens from permanent maxillary molars were treated by applying 38% SDF for 30, 60, or 180 s. Water was applied in the control group. The specimens were immersed in simulated body fluid for 2 weeks, and the mineral precipitation in demineralized dentin was then analyzed using FTIR-ATR, SEM-EDX, and synchrotron radiation X-ray tomographic microscopy (SRXTM). The FTIR-ATR results showed a significant increase in mineral precipitation in the 180 s group after 1 week. However, after 2 weeks, the SRXTM images indicated comparable mineral density between the 30, 60, and 180 s groups. The precipitation of silver chloride and calcium phosphate crystals that occluded dentinal tubules was similar in all experimental groups. In conclusion, an application time of either 30, 60, or 180 s promoted a comparable degree of mineral precipitation in demineralized dentin.

## 1. Introduction

Dental caries remains the most common preventable chronic disease that affects people worldwide. Almost 2.3 billion adults and 532 million children are reported to have untreated dental caries [1]. The treatment of dental caries constitutes a high economic burden in many countries and requires long-term management. Non-invasive or minimally invasive techniques are regarded as cost-effective methods for the management of dental caries [2], and one such method is the use of silver diamine fluoride (SDF) [3,4], which has been reported to prevent 61% [5] and 71% [6] of caries in children and adults, respectively.

The most commonly used SDF products contain 38% SDF (~44,800 ppmF) [7]. SDF solution contains fluoride and silver ions that are stabilized by di-ammine groups, and its main actions are the promotion of remineralization and the inactivation of active carious lesions. Silver ions exert strong antimicrobial effects and reduce collagen degradation by inhibiting dentin collagenases [8,9]. Additionally, silver ions react with phosphate or chloride ions, resulting in the formation of silver salts with low solubility, which hardens soft carious lesions [9]. This subsequently results in a black metallic stain on the tooth surface [10]. The high fluoride concentration in SDF is also intended to promote dentin remineralization through the formation of fluorohydroxyapatite and calcium fluoride [11]. Additionally, the formation of ammonium compounds may have an acid-buffering effect and increase the alkalinity of the environment, which can facilitate the precipitation of mineral apatite [12]. Therefore, the use of SDF reduces further tooth demineralization and facilitates the functional recovery of carious lesions [13].

The application technique of SDF is simple, which makes it suitable for controlling active caries in patients who have special care needs or those who are less cooperative [14]. The application time of SDF ranges from 10 s to 3 min [15], and at least 1–3 min has been suggested to allow the solution to adsorb to the lesions [16,17]. However, the application time of SDF can vary, and it may be shorter than the recommendation when patient cooperation is limited. According to previous studies, the application time may not be related to the success of the treatment [16]. However, the possible reasons or mechanisms that explain this observation are unclear, and supporting evidence is limited. Therefore, the aim of this in vitro study was to assess the effect of different SDF application times (30, 60, and 180 s) on mineral precipitation in demineralized dentin. The null hypothesis in the current study was that the application time does not affect the mineral precipitation in demineralized dentin.

## 2. Material and Methods

### 2.1. Specimen Preparation

The use of extracted human teeth was approved by the Ethics Review Sub-Committee for Research Involving Human Research Subjects of Thammasat University (approval number: 150/2562). In total, 13 extracted third permanent maxillary molars with a similar size and no visible caries were collected from Thammasat University Hospital, Pathum Thani, Thailand. The collected teeth were stored for less than 30 days in 0.1% thymol solution at 23 °C prior to the test.

**Figure 1:**
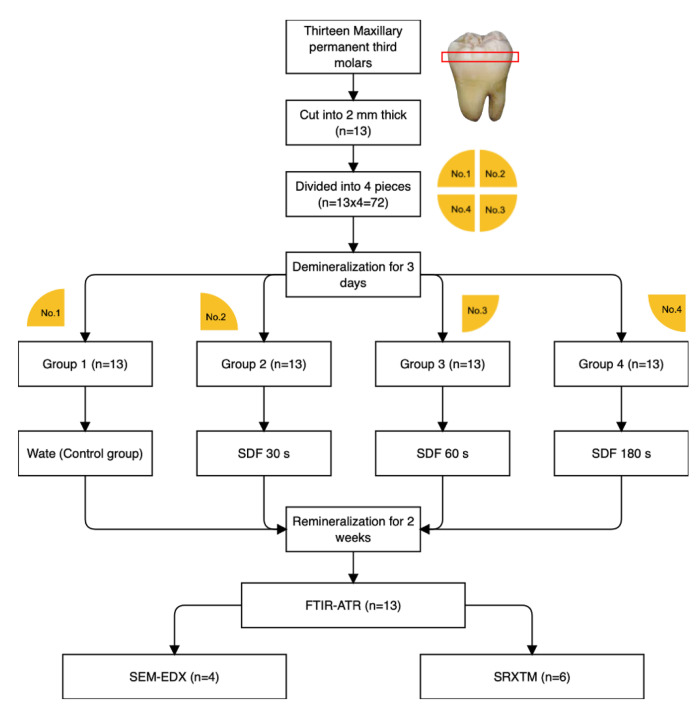
Flowchart representing the methods used in the current study.

Each tooth was cut horizontally and perpendicular to dentinal tubules at ~2 mm below the occlusal surface using a cutting machine (Accutom 50, Struers, Cleveland, OH, USA) to produce dentin slices (2.0 ± 0.1 mm in thickness). Then, the surfaces of the specimens were polished with microfine 4000-grit abrasive papers (Tegramin, Struers, Cleveland, OH, USA) and cleaned with an ultrasonic bath for 5 min. Each dentin slice was then divided into four pieces using a diamond bur to produce a total of 52 dentin specimens. The specimens were demineralized using a simplified method by placing them in a tube containing 10 mL of 17% ethylenediaminetetraacetic acid (EDTA, Faculty of Dentistry, Chulalongkorn University, Bangkok, Thailand) for 72 h to produce mineral-depleted dentin [10,18]. The specimens were rinsed with deionized water and blotted dry. Deionized water (control group) or SDF (253,900 ppmAg and 44,800 ppmF, Topamine^TM^, Dentalife, Victoria, Australia) was applied to specimens from each tooth using the following protocols (*n* = 13/group) (Figure 1).

1. Group 1 (control): 25 μL of deionized water was applied;
2. Group 2 (30 s): 25 μL of SDF was applied and left for 30 s;
3. Group 3 (60 s): 25 μL of SDF was applied and left for 60 s;
4. Group 4 (180 s): 25 μL of SDF was applied and left for 180 s.

The specimens were then rinsed with deionized water for 10 s. They were then placed in a tube containing 5 mL of simulated body fluid (SBF) prepared according to BS ISO 23317:2014 [19]. The SBF solution was prepared by dissolving reagent-grade NaCl (Sigma-Aldrich, St. Louis, MO, USA), NaCO_3_ (Sigma-Aldrich), KCl (Sigma-Aldrich), K_2_HPO_4_·3H_2_O (Sigma-Aldrich), MgCl_2_·6H_2_O (Sigma-Aldrich), CaCl_2_ (Sigma-Aldrich), and Na_2_SO_4_ (Sigma-Aldrich) in deionized water. The pH of the solution was adjusted using tris-hydroxymethyl (Sigma-Aldrich) and 1 M HCl. SBF contains the same phosphate concentration as blood plasma or body fluid (pH = 7.40) [20]. The use of SBF is intended to mimic the oral environment in which SDF adsorbs to dentin when exposed to dentinal fluid. The specimens were incubated at 37 °C for up to 2 weeks without replacing SBF with fresh solution.

### 2.2. Assessment of Mineral Apatite Precipitation Using FTIR-ATR

**Figure 2:**
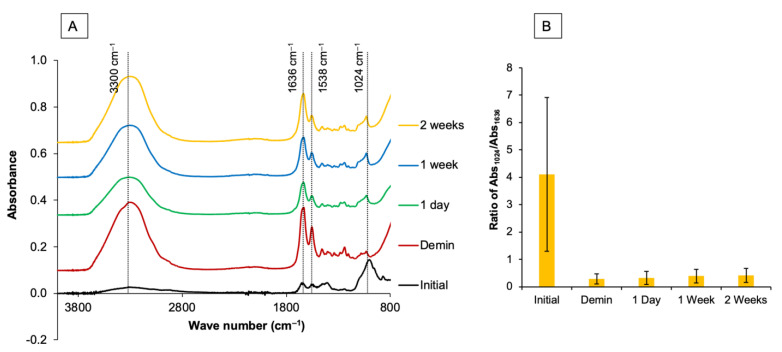
(**A**) Example of FTIR spectra of a specimen from the 60 s group prior to the demineralization process. A strong peak of the phosphate group in mineral apatite (1024 cm^−1^, PO_4_^3−^ stretch) was detected. After demineralization, peaks representing type I collagen, such as 1636 cm^−1^ (C=O stretch, amide I), 1538 (amide II, C-N stretch), and 3300 cm^−1^ (amide A), were stronger than the peak attributable to apatite. (**B**) The area of the FTIR peak at 1024 cm^−1^ versus 1636 cm^−1^ was then obtained to determine the mineral precipitation in dentin.

A Fourier-transform infrared spectrometer (FTIR, Nicolet iS5, Thermo Fisher Scientific, Waltham, MA, USA) equipped with attenuated total reflection (ATR, ID7, Thermo Fisher Scientific) was used to assess the apatite formation on the demineralized dentin using a protocol modified from previous studies (*n* = 13) [21,22,23,24]. FTIR spectra of the specimens before and after immersion in SBF for 1 day, 1 week, and 2 weeks were obtained. The specimens were blotted dry and placed on the ATR diamond. FTIR spectra in the region of 700–4000 cm^−1^ with a resolution of 8 cm^−1^ and 12 repetitions were recorded from the bottom surface (Figure 2A).

Peaks representing mineral apatite (1024 cm^−1^, PO_4_^3−^ stretch) were detected [25]. Furthermore, peaks representing type I collagen in dentin, such as 1636 cm^−1^ (C=O stretch, amide I) [26], 1538 (amide II, C-N stretch) [27], and 3300 cm^−1^ (amide A and amine N-H) [28], were also recorded. The ratio of the FTIR peak area at 1024 cm^−1^ (Abs_1024_), which represents the phosphate group in hydroxyapatite, versus the peak area at 1636 cm^−1^, which represents collagen, was obtained using OMNIC Series software (Thermo Fisher Scientific, Waltham, MA, USA). The mineral/matrix ratio, or Abs_1024_/Abs_1636_, was then calculated (Figure 2B). An increase in the Abs_1024_/Abs_1636_ ratio indicates an increase in mineral precipitation (remineralization) in demineralized dentin.

### 2.3. Assessment of Surface Mineral Precipitation Using SEM-EDX

After immersion in SBF for 2 weeks, representative specimens (*n* = 4) were randomly selected and blotted dry. The specimens were sputter-coated with gold using a sputter-coating machine (Q150R ES, Quorum Technologies, East Sussex, UK) with a current of 23 mA for 45 s. The specimen surface was then examined using a scanning electron microscope (SEM, JSM 7800F, JEOL Ltd., Tokyo, Japan) using an accelerating voltage of 5 kV with a magnification of 2500× to 20,000×. An elemental analysis [29] was performed using a dispersive X-ray spectrometer (EDX, X-Max 20, Oxford Instruments, Abingdon, UK) to analyze the elemental composition (Ca, P, Ag, or F) of the precipitate on representative specimens. The measurement was taken from three different areas of the precipitate with a magnification of 20,000× and a beam voltage set at 5 kV. Data were analyzed using INCA software version 5.05 (ETAS, Stuttgart, Germany).

### 2.4. Assessment of the Degree of Mineral Precipitation (Mineral Density) Using Synchrotron Radiation X-ray Tomographic Microscopy (SRXTM)

After immersion for 2 weeks, the specimens were randomly selected and blotted dry (*n* = 6). Synchrotron radiation X-ray tomographic microscopy (SRXTM) was employed to quantify the amount of mineral precipitation in demineralized dentin. The experiment was carried out at the XTM beamline (BL1.2W: X-ray imaging and tomographic microscopy) at the Synchrotron Light Research Institute (public organization) in Thailand. The synchrotron radiation was generated from a 2.2 Tesla multipole wiggler in the 1.2 GeV Siam Photon Source. For the data collection, each dentin specimen was held in a polyimide tube and mounted on a rotary stage. A total of 900 X-ray projections were obtained at 0–180° with an angular increment of 0.2°. Tomography images were collected with a filtered polychromatic X-ray beam at a mean energy of 14 keV at a distance of 32 m from the source.

The X-ray projections were obtained using a detection system equipped with a 200 μm thick YAG: Ce scintillator, a white-beam microscope (Optique Peter, Lentilly, France), and a pco.edge 5.5 sCMOS camera (2560 × 2160 pixels, 16 bits). All tomographic scans were acquired at a pixel size of 1.44 μm. The X-ray projections were normalized by flat-field correction with open-beam and dark images. The tomograms were reconstructed using Octopus Reconstruction software (TESCAN, Gent, Belgium) [30]. The 3D volume representation was produced using Drishti software [31]. The degree of mineral precipitation was determined from the proportion of dense mineral areas in radiolucent demineralized dentin. Each specimen was measured at 3 different areas chosen at random (~10 × 10 μm). The measurement in each area was performed for 250 slices (250 × 1.44 μm = 360 μm). The mineral density (vol%) was determined by calculating the radiodense areas in the measured volume using Octopus Analysis software (TESCAN, Gent, Belgium).

### 2.5. Statistical Analysis

Data were analyzed using Prism9 for Mac OS (GraphPad Software, San Diego, CA, USA). The normality of data was assessed using the Shapiro–Wilk test. The changes in the Abs_1024_/Abs_1636_ ratio of each group based on the immersion time were analyzed using the Friedman test followed by Dunn’s multiple comparison. The differences in the Abs_1024_/Abs_1636_ ratio of each group were determined using the Kruskal–Wallis test followed by Dunn’s multiple comparison. The mineral density result was analyzed using one-way ANOVA followed by Tukey’s multiple comparisons. All *p* values below 0.05 were considered statistically significant. The post hoc power analysis was performed using G*Power version 3.1.9.6 (University of Dusseldorf, Dusseldorf, Germany). The effect size [32] of each experiment was calculated from the results obtained in the current study, which demonstrated that the sample size used in each test exhibited a power >0.95 at alpha = 0.05.

## 3. Results

### 3.1. Assessment of Mineral Precipitation Using FTIR-ATR

The median Abs_1024_/Abs_1636_ ratios of the control group after 24 h, 1 week, and 2 weeks were comparable (*p* > 0.05) (Table 1). For the 30 s group, the median Abs_1024_/Abs_1636_ ratio increased from 0.282 to 0.308 after 2 weeks. However, the Abs_1024_/Abs_1636_ ratios obtained at each time point were comparable (*p* > 0.05). For the 60 s group, a significant increase in the Abs_1024_/Abs_1636_ ratio was observed after 2 weeks (*p* = 0.0143). For the 180 s group, a significant increase in the Abs_1024_/Abs_1636_ ratio was observed after 1 week (*p* = 0.0029).

At 0 h, the median Abs_1024_/Abs_1636_ ratios of all groups were comparable (Table 1). After 24 h, the median Abs_1024_/Abs_1636_ ratio of the 30 s group was significantly higher than that of the control group (*p* = 0.0461). After 1 week, the median Abs_1024_/Abs_1636_ ratios of both the 30 and 180 s groups were significantly higher than that of 0 h (*p* < 005). Similarly, the median Abs_1024_/Abs_1636_ ratios of both the 30 and 180 s groups after 2 weeks were also significantly higher than that of the control group (*p* < 0.05).

**Figure 3:**
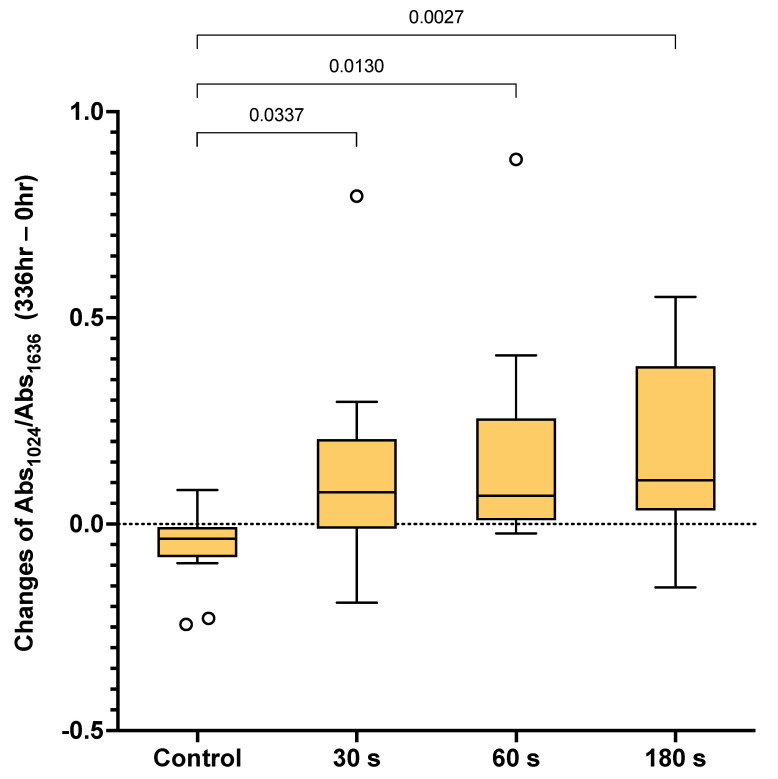
Box plots of the differences in Abs_1024_/Abs_1636_ after 2 weeks compared with the initial value (336 vs. 0 h). The boxes represent the first quartile (Q1) to the third quartile (Q3), the horizontal lines in the box represent the median, and the whiskers represent the maximum and minimum values (*n* = 13). The line indicates *p* < 0.05. Circles are outliers.

The changes in the Abs_1024_/Abs_1636_ ratio from 0 h to 2 weeks in each group are demonstrated in Figure 3. The Abs_1024_/Abs_1636_ ratio obtained from the control group (−0.035) was significantly lower than that of the 30 (0.077) (*p* = 0.0337), 60 (0.068) (*p* = 0.0130), and 180 s (0.106) (*p* = 0.0027) groups. However, the values obtained from the 30, 60, and 180 s groups were comparable (*p* > 0.05).

### 3.2. Assessment of Surface Mineral Precipitation Using SEM-EDX

**Figure 4:**
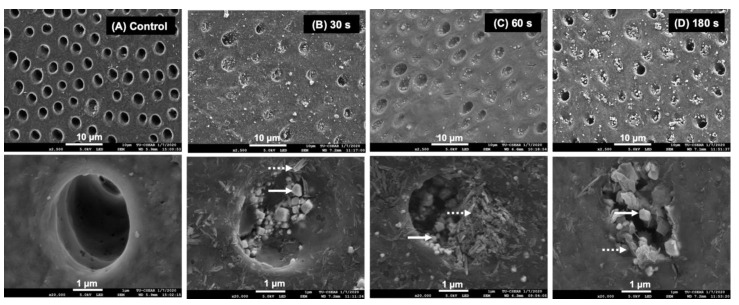
SEM images at low and high magnification of a representative specimen from each group after 2 weeks. (**A**) The surface of the specimen from the control group showed no mineral precipitation. Precipitation occluding dentinal tubules was detected in the specimens from the (**B**) 30, (**C**) 60, and (**D**) 180 s groups. Irregular shapes (solid arrows) and needle-like precipitates (dash arrows) were observed.

**Figure 5:**
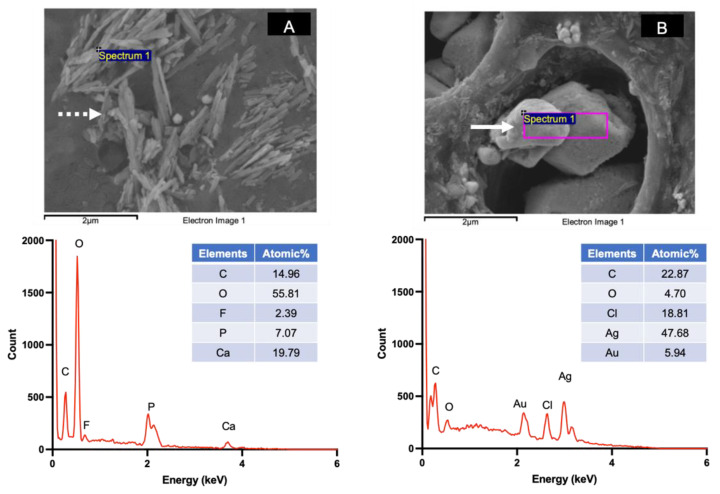
Examples of precipitated crystals and EDX spectra detected on the specimen surface in the 30, 60, and 180 s groups. (**A**) The calcium phosphate precipitate (needle shape, dashed arrow) containing Ca, F, and P. (**B**) Crystals containing Ag and Cl (irregular shape, solid arrow).

The SEM images of specimens treated with water showed patent dentinal tubules without mineral precipitation (Figure 4A). The peritubular dentin and dentinal tubules of specimens from the 30, 60, and 180 s groups were filled with precipitated crystals (Figure 4B–D). Precipitates containing Ca, P, and F were detected (Figure 5A). Additionally, crystals consisting of Ag and Cl were also observed (Figure 5B).

### 3.3. Assessment of the Degree of Mineral Precipitation (Mineral Density) Using Synchrotron Radiation X-ray Tomographic Microscopy (SRXTM)

**Figure 6:**
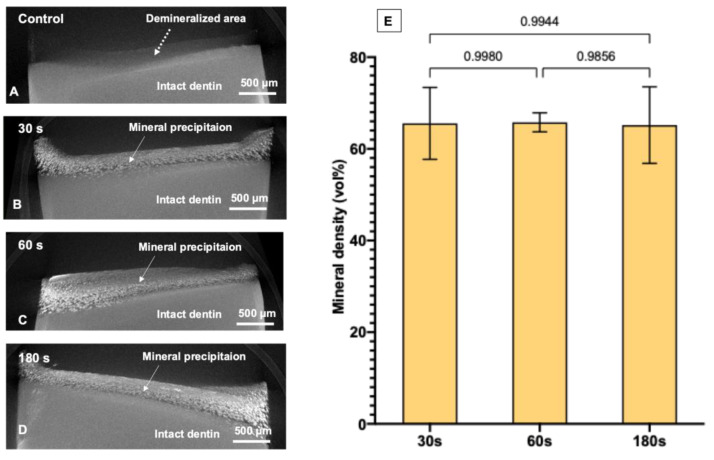
Reconstructed SRXTM images of a representative specimen from each group after immersion in simulated body fluid for 2 weeks. (**A**) The control group showed a demineralized area (~250 μm depth, dashed arrow) without evident mineral precipitation. (**B**–**D**) Reconstructed images from the representative specimen from 30, 60, and 180 s groups, respectively. The images show substantial mineral precipitation in the demineralized area (solid arrows). (**E**) Mean mineral density obtained from 30, 60, and 180 s groups. Error bars are 95% CI (*n* = 6). Lines indicate *p* > 0.05. Data from the control group were not analyzed because mineral precipitation was not detected. The 3D visualization images of the specimens are provided in the Supplementary Materials (S1, S2).

The reconstructed 3D volume of the specimens from SRXTM images included a radiolucent area (thickness of ~250 μm), which was inferred to be the area of fully demineralized dentin (Figure 6A). However, the demineralized layer of specimens treated with SDF contained dispersed radiodense clusters, which were inferred to be precipitated minerals (Figure 6B–D). For the control group, the demineralized layer showed no precipitation (0 vol%). The degree of mineral precipitation (mineral density, vol%) of the 30, 60, and 180 s groups was 65.6 ± 7.5 vol%, 65.8 ± 2.0 vol%, and 65.2 ± 8.0 vol%, respectively (Figure 6E). The results of the 30, 60, and 180 s groups were all comparable (*p* > 0.05). Videos of the 3D visualization of representative specimens from the control group and the SDF-treated groups are provided in the Supplementary Materials (Videos S1 and S2).

## 4. Discussion

SDF is a cost-effective method for controlling the progression of dental caries. However, patients requiring special care due to mental, developmental, or physical disabilities may exhibit very limited cooperation during preventive oral health care practices [14], which may affect the clinical use of the material in these patients. Different studies have recommended application times that range from 30 s to 3 min [15,17]. Although it has been suggested that the duration of SDF application in carious lesions does not directly correlate with the success of the treatment [16], an explanation for this observation is still lacking. Hence, the current study aimed to assess the effect of applying SDF for different times (30, 60, or 180 s) on the mineral precipitation of demineralized dentin.

The results obtained after 1 week showed that the longest SDF application time (180 s) enabled a rapid increase in mineral precipitation. However, FTIR-ATR, SEM-EDX, and SRXTM results obtained after 2 weeks revealed that mineral precipitation was comparable between the 30, 60, and 180 s groups. Hence, the null hypothesis was partially rejected.

The use of simulated body fluid is intended to mimic dentinal fluid in dentin. Simulated body fluid is a solution that is supersaturated with respect to apatite [33]. However, mineral precipitation was only detected in the specimens treated with SDF (Figure 3, Figure 4 and Figure 5). A possible explanation is that SDF encourages the adsorption of Ca and P, resulting in mineral apatite formation [7,34]. Additionally, the pH of most SDF products is in the alkaline range [35]. This may facilitate suitable conditions [12] for the precipitation of low-solubility fluorohydroxyapatite [36]. SDF also helps to preserve collagen, which acts as a scaffold for mineral precipitation [8,13]. Hence, the increase in SDF application time may help to facilitate mineral precipitation in demineralized dentin. This may explain why a significant increase in the Abs_1024_/Abs_1636_ ratio was detected in the 180 s group before it was observed in the other groups. However, the ratios measured for the 30, 60, and 180 s groups at each time point were not significantly different in the current study. This may suggest that an application time of 30–180 s allows for sufficient SDF adsorption to dentin and leads to comparable remineralization. This could potentially help to reduce the concerns of clinicians when they need to apply SDF in challenging situations. It should be noted that the current study only employed FTIR to assess mineralization. The use of XPS or XRD may be needed to characterize mineral precipitation in demineralized dentin.

The SEM images of representative specimens from the 30, 60, and 180 s groups after 2 weeks demonstrated similar patterns of mineral precipitation in both the intra-tubular and peri-tubular dentin of the specimens. It has been reported that the primary mineral precipitation after SDF application is formed by calcium phosphates and silver salts [9,37]. Crystals containing Ca and P were detected in specimens from the experimental groups in the current study, and the precipitation of these calcium phosphate crystals may have contributed to the increase in the FTIR peak ratio. It has also been suggested that fluoride ions from SDF might substitute hydroxyl groups, producing low-solubility and acid-resistant fluorohydroxyapatite [11]. Crystals containing Ca, P, and F were also detected. In the current study, crystals containing fluoride were not generally detected, which is in accordance with the findings from previous studies [8,9,38]. A possible explanation may be that the fluoride level was lower than the detection limit of EDX [9,39].

It has been proposed that the formation of silver salts potentially acts as a protective layer against dental caries and blocks dentinal tubules to protect the pulp–dentin complex [7,11]. The EDX results suggest that the metallic crystals that precipitated on the specimen surface may be AgCl salts [37]. The reaction between SDF and water produces highly soluble silver phosphate (solubility of 6.4 × 10^−3^ g/100 mL) and silver oxide (1.3 × 10^−3^ g/100 mL), as shown in Equation (1).

2Ca_5_(PO_4_)_3_OH + 20Ag^+^ → 2Ag_3_PO_4_ + 10Ca^2+^ + Ag_2_O + H_2_O (1)

Silver phosphate and silver oxide can readily react with chloride in an alkaline solution to form silver chloride with lower solubility (solubility of 8.9 × 10^−5^ g/100 mL) [7]. Hence, the precipitation of AgCl has been commonly reported in published studies [8,11], which is in agreement with the current study. Additionally, the application of SDF is hypothesized to encourage the formation of CaF_2_, which can act as a fluoride reservoir [11,40]. However, the formation of CaF_2_ globules was not detected on specimens in the current study. A possible explanation may be that CaF_2_ was quickly washed away upon rinsing [11,41].

SDF has been shown to promote mineral precipitation in lesions of up to 150 μm [42], and the increase in mineral density has helped to reduce the lesion depth from ~250 μm to ~100 μm [43]. The current study showed that the mineral density in demineralized dentin was increased from 0 vol% (control group) to ~66 vol% after SDF application. However, SDF application times of 30, 60, and 180 s showed comparable degrees of mineral precipitation after 2 weeks. This is in agreement with the results of the FTIR studies. The results also showed that high-radiodensity minerals precipitated throughout the entire depth of the demineralized layer (~250 μm). However, the precipitation was denser on the outer surface than in the inner area, which could be due to the shrinkage of dentin (Figure 6B–D and Video S2). It is noted that synchrotron radiation X-ray tomographic microscopy (SRXTM) was employed in the current study to quantify the degree of mineral precipitation (mineral density). The benefit of the SRXTM technique is the higher resolution of images (pixel size ~1.44 μm) compared with conventional micro-CT (pixel size ~8 μm) [44]. However, silver is highly radiopaque, which could potentially lead to the overestimation of the mineral density in the demineralized area [45]. Furthermore, shrinkage of dentin was detected after SDF application, which may have affected the measurements. Additionally, precipitated crystals that were smaller than the pixel size (<1.44 μm) may not have been detected by SRXTM. Hence, the actual degree of mineral precipitation may be different from the calculated values.

The results of the current study may partially explain the lack of correlation between the SDF application time and clinical outcomes [16]. This may be beneficial when treating patients with limited cooperation. However, this was an in vitro study, which cannot mimic the oral environment. In future research, it would be of interest to determine the effect of different application times with a much shorter duration, such as 5–10 s, on the degree of mineral precipitation. Additionally, the different application times may affect the degree of ion penetration into the pulp-dentin complex, which could induce cytotoxic effects or changes of behavior of dental pulp cells [46,47]. This could be another important aspect for future studies.

## 5. Conclusions

An SDF application time of 180 s was associated with a rapid increase in mineral apatite precipitation after 1 week. However, after 2 weeks, the degree of mineral precipitation with different SDF application times (30 s, 60 s, or 180 s) was comparable.

## Figures and Tables

**Table 1:** The Abs_1024_/Abs_1636_ ratios (median, min–max) obtained from specimens in each group for up to 2 weeks (*n* = 13). Lower- and uppercase letters within the same row and same column, respectively, indicate significant differences (*p* < 0.05).

Groups/ Time	0 h Median (Min,Max)	24 h Median (Min,Max)	1 Week Median (Min,Max)	2 Weeks Median (Min,Max)	Friedman Statistics	*p* Values
Control (Water)	0.134 (0.070,0.453)	0.133 (0.07,0.35) ^A^	0.123 (0.07,0.38) ^B,C^	0.117 (0.07,0.38) ^D,E^	11.34	>0.05
SDF 30 s	0.282 (0.080,0.722)	0.244 (0.093,0.928) ^A^	0.318 (0.101,0.949) ^B^	0.308 (0.104,0.935) ^D^	5.215	>0.05
SDF 60 s	0.116 ^a^ (0.068,0.613)	0.173 (0.040,0.593)	0.148 (0.084,0.804)	0.206 ^a^ (0.088,0.957)	0.48	^a^ 0.0143
SDF 180 s	0.180 ^a,b^ (0.048,0.421)	0.202 ^c,d^ (0.061,0.701)	0.316 ^a,c^ (0.093,0.832) ^C^	0.250 ^b,d^ (0.126,0.895) ^E^	20.54	^a^ 0.0029 ^b^ 0.0085 ^c^ 0.0085 ^d^ 0.0234
Kruskal–Wallis statistics	2.667	8.021	12.80	16.12		
*p* values	>0.05	^A^ 0.0461	^B^ 0.168 ^C^ 0.0083	^D^ 0.0023 ^E^ 0.0046		

## Data Availability

The datasets generated during and/or analyzed during the current study are available from the corresponding author on reasonable request.

## Supplementary Materials

The following are available online at https://www.mdpi.com/article/10.3390/dj9060070/s1, Video S1: Representative specimen of the control group showed no obvious mineral precipitation. Video S2: Representative specimen of the experimental groups showed mineral precipitation on the surface and inside the demineralized area. The precipitation is indicated by green.
